# High‐Temperature Single‐Photon Emission From Covalently Functionalized van der Waals Heterostructures

**DOI:** 10.1002/advs.202511319

**Published:** 2025-10-07

**Authors:** S. Carin Gavin, Hsun‐Jen Chuang, Anushka Dasgupta, Moumita Kar, Kathleen M. McCreary, Sung‐Joon Lee, M. Iqbal Bakti Utama, Chunxi Zhou, Xiangzhi Li, George C. Schatz, Tobin J. Marks, Mark C. Hersam, Berend T. Jonker, Nathaniel P. Stern

**Affiliations:** ^1^ Department of Physics and Astronomy Northwestern University Evanston IL 60208 USA; ^2^ Materials Science and Technology Division United States Naval Research Laboratory Washington D.C. 20375 USA; ^3^ Department of Materials Science and Engineering Northwestern University Evanston IL 60208 USA; ^4^ Department of Chemistry Northwestern University Evanston IL 60208 USA; ^5^ Department of Physics Stevens Institute of Technology Hoboken NJ 07030 USA; ^6^ Center for Quantum Science and Engineering Stevens Institute of Technology Hoboken New Jersey 07030 USA; ^7^ Department of Chemical and Biological Engineering Northwestern University Evanston IL 60208 USA; ^8^ Materials Research Center Northwestern University Evanston IL 60208 USA; ^9^ Department of Electrical and Computer Engineering Northwestern University Evanston IL 60208 USA

**Keywords:** covalent functionalization, defect emission, diazonium, quantum emitter, transition metal dichalcogenides, tungsten diselenide, 2D materials

## Abstract

Two‐dimensional (2D) transition metal dichalcogenides (TMDs) such as tungsten diselenide (WSe_2_) are attractive nanomaterials for quantum information applications due to single‐photon emission (SPE) from intrinsic atomic defects. Defect and strain engineering techniques have been developed to produce high purity, deterministically placed SPE in WSe_2_. However, a major challenge in the application of these techniques is the low temperature required to observe defect‐bound TMD exciton emission, typically limiting SPE to *T* < 30 K. SPE at higher temperatures either loses purity or requires integration into complex devices such as optical cavities. Here, 2D heterostructure engineering and molecular functionalization are combined to achieve high purity (>90%) SPE in strained WSe_2_ persisting to over *T* = 90 K. Covalent diazonium functionalization of graphite in layered WSe_2_/graphite heterostructures maintains high purity up to *T* = 90 K and single‐photon source integrity up to *T* = 115 K. This method preserves the best qualities of SPE from WSe_2_ while increasing working temperature to more than three times the typical range. This work demonstrates the versatility of surface functionalization and heterostructure design to synergistically improve the properties of quantum emission and offers new insights into the phenomenon of SPE from 2D materials.

## Graphical abstract

Covalent diazonium functionalization of graphite‐WSe_2_ heterostructures produces high‐purity single‐photon emission from WSe_2_ at *T* = 90 K, far above the typical *T* = 10 K limit. The chemical modification reshapes graphite's electronic structure and couples to WSe_2_ midgap states, stabilizing quantum emitters at elevated temperatures and opening pathways toward practical, thermally robust 2D single‐photon devices.

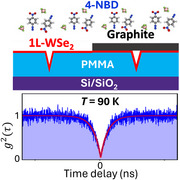

## 1 Introduction

Single‐photon emission (SPE) from solid‐state sources is valuable for quantum information applications because of its scalability and integration into optoelectronic, photonic, and quantum architectures. Various solid state SPE platforms, such as carbon nanotubes, embedded quantum dots, and crystalline defects, each bring distinct advantages and drawbacks in terms of desirable SPE characteristics like purity, brightness, indistinguishability, deterministic creation, and high working temperatures.^[^ ^1^ ^]^ Despite this panoply, a universally ideal source is not evident, and the specific properties of diverse materials provide opportunities to explore distinct physical quantum systems and optimize SPE for different applications. For example, SPE from atomic‐scale defects in two‐dimensional (2D) van der Waals materials including transition metal dichalcogenides (TMDs) and hexagonal boron nitride leverages unique advantages of 2D materials to quantum optical phenomena, such as the customization of layered heterostructures and the accessibility of electrons in a 2D surface.

Within the TMD class of materials, monolayer tungsten diselenide (WSe_2_) is a uniquely prolific host of SPE, with the distinct advantage of deterministically placing emitters using mechanical strain. After early investigation of this phenomenon showed that SPE from WSe_2_ monolayers was detected at edges or folds,^[^ ^2^, ^3^, ^4^, ^5^ ^]^ a myriad of techniques have been developed to harness strain and create bright on‐demand emitters.^[^ ^5^, ^6^, ^7^, ^8^ ^]^ In addition to strain engineering, techniques have developed to improve other qualities of TMD SPE such as line width and purity for better integration into functional quantum systems.^[^ ^1^, ^9^, ^10^, ^11^ ^]^ Despite these advances in material control, viable SPE from TMDs remains challenging. The spectral energy of emitters is largely uncontrolled, spectral crowding from strain‐activated defect emission can adversely affect photon purity, and the observation of SPE is typically restricted to low cryogenic temperatures. The common atomic defects native to WSe_2_ create defect‐bound transitions with very low binding energies, meaning that SPE is generally limited to less than *T* = 30 K,^[^ ^2^, ^12^, ^13^ ^]^ with the highest purity (over 90%) restricted to less than *T* = 15 K.^[^ ^9^, ^14^, ^15^ ^]^ SPE that can be sustained to higher temperatures either lose purity^[^ ^16^ ^]^ or require complex fabrication such as integration into optical cavities^[^ ^17^ ^]^ or defect engineering by electron beam irradiation to create defect states deeper within the bandgap.^[^ ^18^ ^]^ Although strain engineering in TMDs has been robustly explored for controlling SPE, the 2D nature of monolayer TMDs provides additional avenues for optimization that have only recently been exploited. At low temperatures, layering graphite onto strained WSe_2_ drastically improves SPE purity by quenching nearly all free and bound exciton emission.^[^ ^11^ ^]^ Similarly, chemical functionalization, already established as a versatile tool for the modification of nanomaterials,^[^ ^19^, ^20^, ^21^, ^22^, ^23^ ^]^ can exploit the accessible surface of 2D materials to improve spectral isolation and purity of SPE in strained WSe_2_.^[^ ^10^ ^]^ The interactions and bonding involved in the heterostructures and chemical functionalization are distinct, suggesting that these approaches could be synergistic, but the combination of these material approaches for modifying SPE has not yet been investigated. In this work, we harness these two powerful tools for manipulating 2D materials to enhance SPE properties: heterostructure engineering and surface modification. The layering of graphite on nano‐indented WSe_2_ creates high purity (>90%) SPE by suppressing competing radiative emission channels through ultra‐fast charge transfer.^[^ ^11^ ^]^ Functionalization of this heterostructure with nitrobenzene diazonium tetrafluoroborate (4‐NBD) results in covalent molecular bonds on the graphite surface, which introduces a bandgap to the semi‐metal that interacts with mid‐gap defect states in WSe_2_. The resulting bright and pure SPE persists up to *T* = 115 K, more than triple the typical limit of similarly pure SPE in WSe_2_. Mechanisms of this high‐temperature SPE are discussed from both experimental and computational perspectives. These results demonstrate tailored SPE at a valuable intersection of determinism, purity, intensity, and temperature, and they present an effective approach for understanding and manipulating quantum emission in layered TMD heterostructures.

## 2 Results

### 2.1 4‐NBD Functionalization of Graphite/WSe_2_ Heterostructures for SPE at 90 K

**Figure 1:**
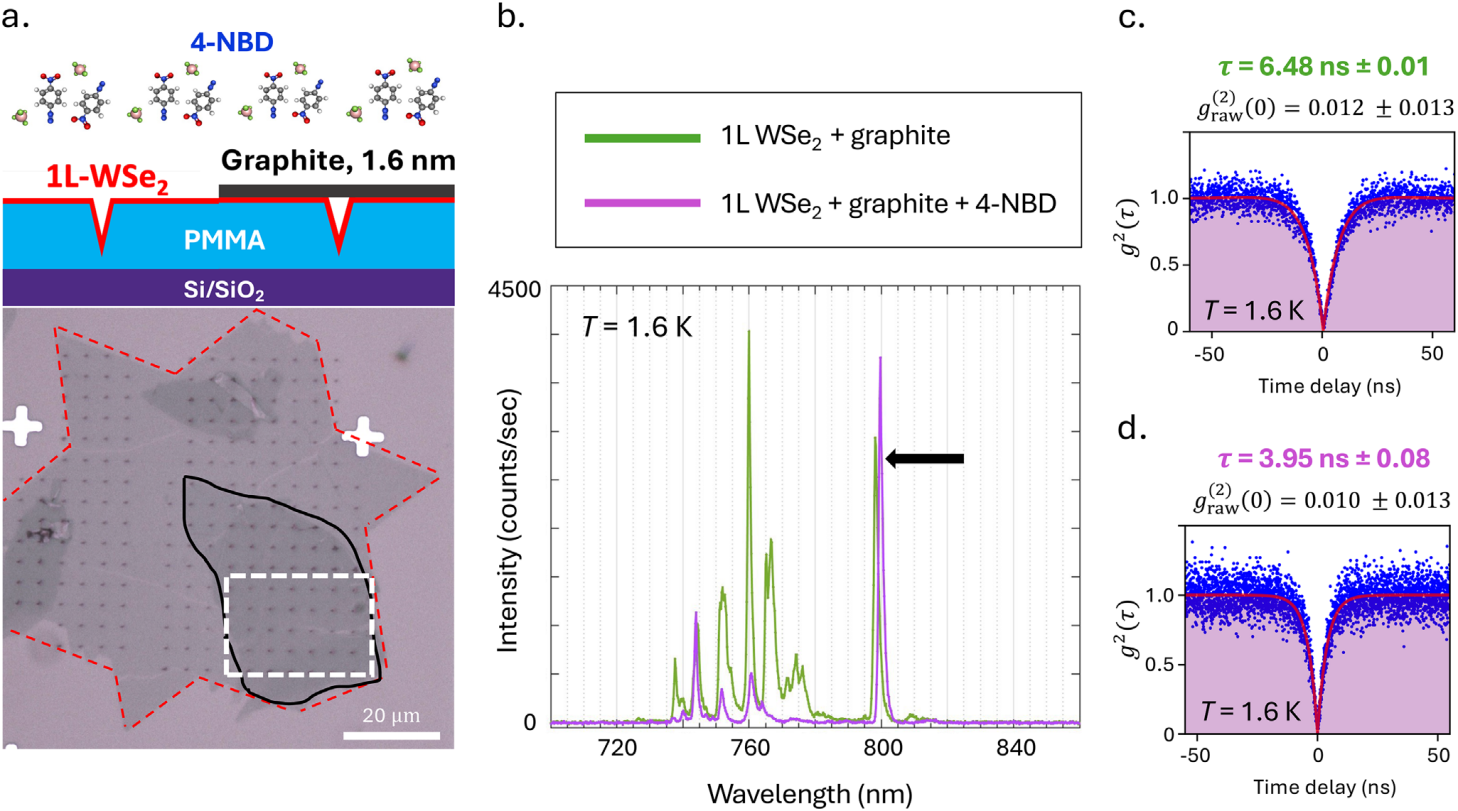
a) Optical microscope image and simplified side‐view schematic of the functionalized heterostructure. Detailed indent geometry is discussed in Section 2.3 and in the Supporting Information. A layer of polymethyl methacrylate (PMMA) is spin‐coated onto a dielectric substrate of silicon/silicon dioxide (Si/SiO_2_). Monolayer (1L) WSe_2_ (red) is then transferred onto PMMA and indented with atomic force microscopy (AFM). Graphite (black) is then transferred over part of this monolayer. The white dashed box outlines the area between graphite and WSe_2_ that has been nano‐squeegeed together. This entire heterostructure is functionalized with 4‐NBD, which leaves nitrophenyl groups either covalently or non‐covalently adsorbed to the surface. b) Spectra of the same squeegeed graphite‐covered indent location before (green) and after (purple) functionalization, showing an increase in emitter intensity of the narrow emission feature at 800 nm for the same amount of laser excitation power. The laser excitation wavelength used here and throughout the work is 532 nm. c) *g* ^(2)^(τ) measured for the emitter highlighted in (b) prior to functionalization. The emitter lifetime is τ = 6.48 ± 0.01 ns. d) *g* ^(2)^(τ) measured for the same emitter after functionalization, where the lifetime is now τ = 3.95 ± 0.08 ns, a 40% decrease in emitter lifetime. The fit parameters to extract the recombination lifetime are discussed in Section I (Supporting Information).

Previous work has shown that both 4‐NBD functionalization^[^ ^10^ ^]^ and heterostructure engineering with graphite^[^ ^11^ ^]^ improve the purity of SPE from monolayers of WSe_2_. These outcomes were a result of physisorption of molecules onto WSe_2_ and the van der Waals interactions between WSe_2_ and graphite, respectively. As such, both methods represent SPE modification based on weak electrostatic forces. Yet more powerful modification to the electronic structure comes from systems that have strong chemical, or covalent, interactions. Diazonium compounds are known to covalently functionalize carbon‐based systems, such as single‐walled carbon nanotubes^[^ ^21^ ^]^ and graphene/graphite.^[^ ^19^, ^20^, ^24^, ^25^, ^26^, ^27^, ^28^ ^]^ Based on this precedent, the functionalization dynamics in a WSe_2_/graphite heterostructure should be distinct from that of WSe_2_ alone. To explore this possibility and its effect on quantum emission, graphite with thickness of 1.6 nm, or about 5 layers, is transferred onto a nanoindented monolayer of WSe_2_ grown by chemical vapor deposition (CVD) (**Figure** **1**a). The heterostructures are prepared following the method of Ref. [11], with strain introduced using a nanoindentation array and the layers “nanosqueegeed” to remove impurities between them and improve contact quality, according to the precedent established by Rosenberger et al.^[^ ^16^, ^29^ ^]^ This hybrid method consistently produces large monolayer areas and a reliably high yield of SPE from the strain array,^[^ ^16^ ^]^ both of which are valuable for the scalability of quantum emission since monolayer area achieved by micromechanical exfoliation is variable and much smaller on average.^[^ ^30^, ^31^ ^]^ The heterostructure is then functionalized by being submerged in an aqueous solution of 4‐NBD. This process dopes the materials present since 4‐NBD is electrophilic, and nitrophenyl (NPh) radicals form as a result. These radicals may either bond to the surface covalently or self‐react, forming chains of multiple nitrophenyls (oligomers), as described in previous work.^[^ ^10^ ^]^ Further details of sample fabrication are found in Section 4.

Figure 1b shows the effect of functionalization on SPE from the WSe_2_/graphite heterostructure at low temperature. For the same graphite‐covered indent location at *T* = 1.6 K as measured with the same amount of laser excitation power, many higher‐energy (below ∼780 nm) localized emitters are quenched by 4‐NBD, whereas the intensity of the emitter at 800 nm is increased by functionalization. Figure 1c,d shows *g* ^(2)^(τ) measurements for this emitter, demonstrating that it is SPE of high purity above 90%. The lifetime of SPE extracted from the correlation relaxation from τ = 0 is reduced by functionalization from τ = 6.48 ns with graphite alone to τ = 3.95 ns after functionalization. The impact of 4‐NBD functionalization on the temporal dynamics of SPE in WSe_2_/graphite heterostructures is a strong indication that this structure has changed the mechanisms underlying the phenomenon.

**Figure 2:**
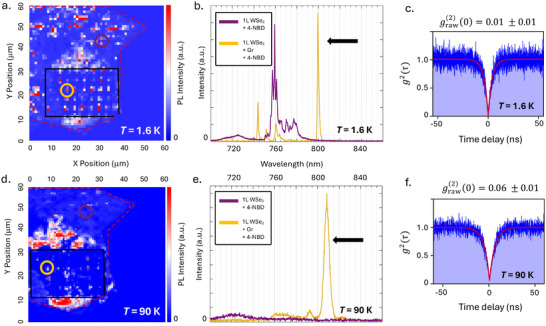
Results of a WSe_2_/graphite heterostructure functionalized with 4‐NBD; here the graphite thickness is 1.6 nm. a) Emission intensity map of the heterostructure at *T* = 1.6 K. The red dashed line outlines the WSe_2_ area. The black dashed box within this area outlines the portion of graphite and WSe_2_ that are squeegeed together. The relatively bright emission surrounding the squeegeed area is due to the flourescence of residue such as transfer polymer that was pushed out from between the layers.^[^ ^29^ ^]^ b) Spectrum of two indent locations, one where just WSe_2_ is functionalized with 4‐NBD (purple) and one where the graphite heterostructure is functionalized (yellow). The yellow spectrum has a strong emission line around 800 nm, which is shown to be SPE at low temperature in (c). Panels (d–f) show the same data at *T* = 90 K. In (e), the purple spectrum of the same indent as (b) shows that localized emission on the functionalized WSe_2_ is gone, whereas strong emitters remain on the graphite heterostructure side (yellow). This emitter is still high purity SPE even at *T* = 90 K.

To highlight that both 4‐NBD and graphite contribute to changed dynamics, **Figure** **2** compares the low and high temperature performance of SPE from the functionalized heterostructure to the plain WSe_2_ functionalized with 4‐NBD. At low temperatures (*T* = 1.6 K), the results are reliable: sharp, localized emission is observed from indent locations (Figure 2a–c). The SPE spectra from indented WSe_2_ (dark purple) are consistent with previous reports of 4‐NBD functionalization of strained WSe_2_, showing a red‐shifted exciton and suppression of most excitonic emission.^[^ ^10^ ^]^ The graphite‐covered indent (the same as that shown in Figure 1b) exhibits relatively more quenching of excitonic features, and fewer isolated emission lines remain, but the high purity SPE remains at 800 nm (yellow) (Figure 2c).

Although the results for *T* = 1.6  K are in line with previous expectations, the combination of graphite and 4‐NBD surprisingly sustains the high‐purity SPE to much higher temperatures. Figure 2d–f shows the properties of the same indent locations (circled in purple and yellow) at *T* = 90 K. At this temperature, sharp emission from the plain WSe_2_ indent has disappeared, in accordance with the typical temperature restrictions of defect‐bound emission in TMDs. In contrast, intense sharp features persist on the graphite‐covered indents. These features are once again shown to be SPE with purity over 90%. In addition to high purity, the peak intensity remains high at *T* = 90 K with a maximum value of approximately 800 counts/sec, resulting in an excellent signal‐to‐noise ratio in the measurements *g* ^(2)^(τ) (Figure 2f). Although this is the cut‐off temperature for purity over 90%, the emitter remains viable with *g* ^(2)^(0) < 0.5 up to *T* = 115 K and detectable in the spectrum to *T* = 150 K, on par with other methods producing higher temp SPE in WSe_2_.^[^ ^17^, ^18^ ^]^ A comparison of temperature, purity, and emitter lifetime is shown in Figure S6 (Supporting Information), and the full temperature evolution is presented in Section 3.

Not all SPE observed on the functionalized heterostructure at low temperatures remains up to *T* = 90 K. Rather, localized emission features that persist to higher temperatures lie within a narrower wavelength range than those visible at low temperatures. However, the yield of these emitters across the strain array is high; the ratio of candidate SPE at low temperatures which persist to higher temperatures is about 80%, discussed in Figure S3 (Supporting Information). Figure S5 (Supporting Information) details some of these additional emission spectra and correlation measurements confirming SPE from graphite‐covered indents present in Figure 2d. The high‐intensity emission peaks at this temperature are centered between 780 and 820 nm, whereas for *T* < 10 K, SPE are commonly anywhere between 720 and 820 nm.^[^ ^5^, ^6^, ^10^, ^14^ ^]^ In other words, lower‐energy SPE transitions selectively last to higher temperatures, while higher‐energy SPE transitions are quenched at the typical temperature scales. This behavior is similar to the effect of 4‐NBD functionalization at low temperatures, where higher energy emission is suppressed after functionalization, but the lower energy SPE intensity is increased (Figure 1b).

Lastly, the effect of graphite thickness on the maximum SPE temperature was tested. To do this, a sample of identical structure but thicker graphite (∼8 nm) was measured. Detailed in Figure S7 (Supporting Information), these results show that a sharp emitter at the same wavelength (800 nm) is also high purity SPE at elevated temperature. However, the maximum temperature is over 30 K lower than that of the thinner graphite. While SPE with thin graphite persists to around *T* = 115 K, the purity and intensity of those with thicker graphite are lost around 70–80 K. From these experimental results, maximum SPE temperature and graphite thickness are correlated, the details of which are discussed in following sections.

### 2.2 Covalent Functionalization of Graphite

**Figure 3:**
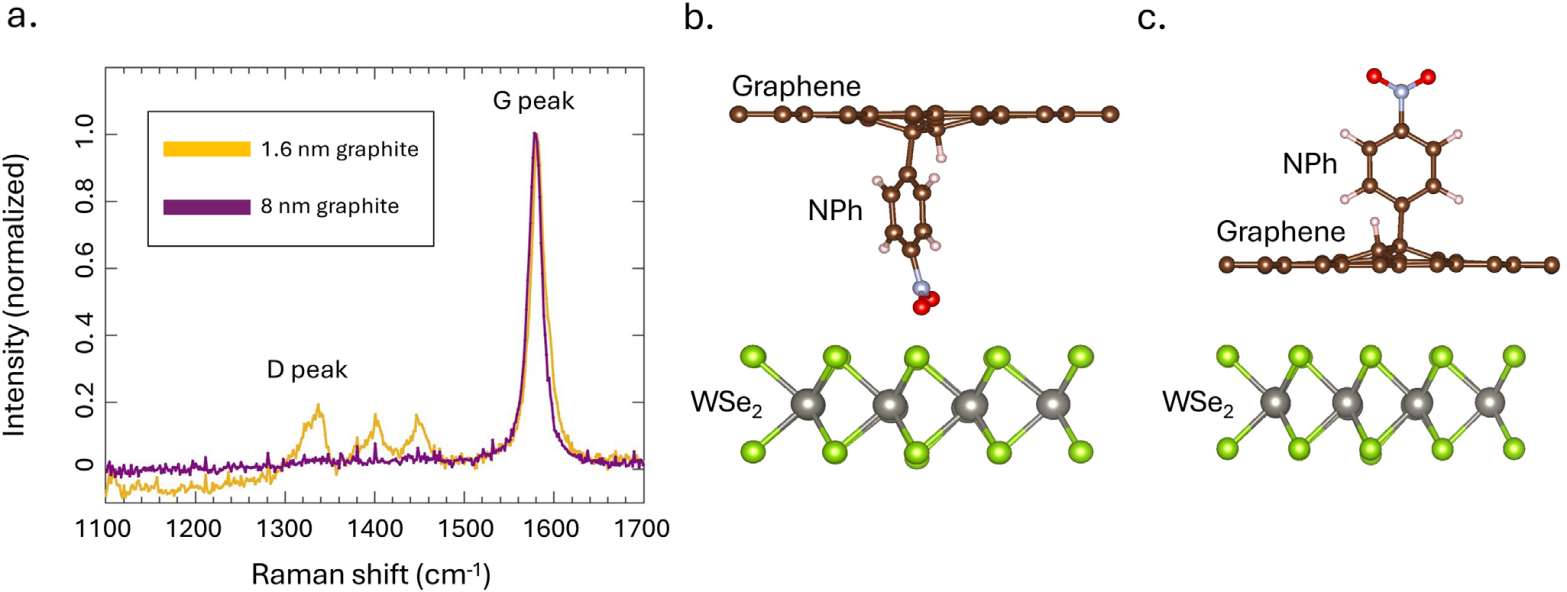
a) Raman spectra of graphite after 4‐NBD functionalization, one with 1.6‐nm thickness (yellow) and the other with 8 nm thickness (purple). Both spectra are normalized to the graphite G peak. The D peak is detectable on the thin graphite with a resulting D/G peak ratio of about 20%, indicating disruptions on the graphite surface due to covalent bonds. b,c) Images of possible nitrophenyl (NPh) position in layered heterostructure configurations. In (b), NPh is covalently attached to graphene but located between the graphene and WSe_2_. In (c), NPh is covalently bonded to the top of the graphene. This configuration is more stable and consistent with experimental findings from (a).

Following the novel results discussed above, Raman spectroscopy was used to assess the nature of the 4‐NBD functionalization and its interaction with the 2D layers in the heterostructure. Pristine graphite has a characteristic Raman feature centered around 1580 cm^−1^ called the “G peak”;^[^ ^32^ ^]^ when there are disturbances in the carbon bonds on the surface as a result of covalent functionalization, a new Raman mode appears around 1350 cm^−1^, known as the “D peak”.^[^ ^19^ ^]^ The ratio of these two peaks (D/G) characterizes the degree of covalent functionalization on the graphite surface. **Figure** **3**a shows the Raman spectra of the two functionalized heterostructures presented in Section 2.1, one in which graphite is approximately 8 nm thick, and the other in which graphite is 1.6 nm thick. Thin graphite should have a higher degree of covalent functionalization than thick graphite overall given the lower density of states.^[^ ^19^ ^]^ Here, the D peak is not detected on the thick graphite. However, for the thinner graphite, the D/G peak ratio is approximately 20%, referenced with the primary D peak at 1350 cm^−1^. Two additional Raman peaks are observed around 1400 and 1450 cm^−1^, which can indicate other types of lattice disruptions. This confirms that our specific experimental heterostructure has covalent bonds between NPh and graphite from the functionalization process, which facilitates the observation of SPE up to ∼115 K. This now correlates the maximum SPE temperature with the degree of covalent functionalization.

Figure 3b,c shows schematics of two possible heterostructure arrangements: one in which the nitrophenyl group is mainly between WSe_2_ and graphene and one in which it is mainly on top of graphene. To support the interpretation that 4‐NBD preferentially functionalizes the top surface of graphite, calculations were performed to find the most energetically stable chemical configuration of these elements. The total energy of surface functionalization when NPh is on top of graphene is lower by 2.62 eV compared to between layers, indicating that it is more stable. This calculation combined with the Raman spectra reveal that the arrangement in Figure 3c is the likely configuration of the heterostructures.

**Figure 4:**
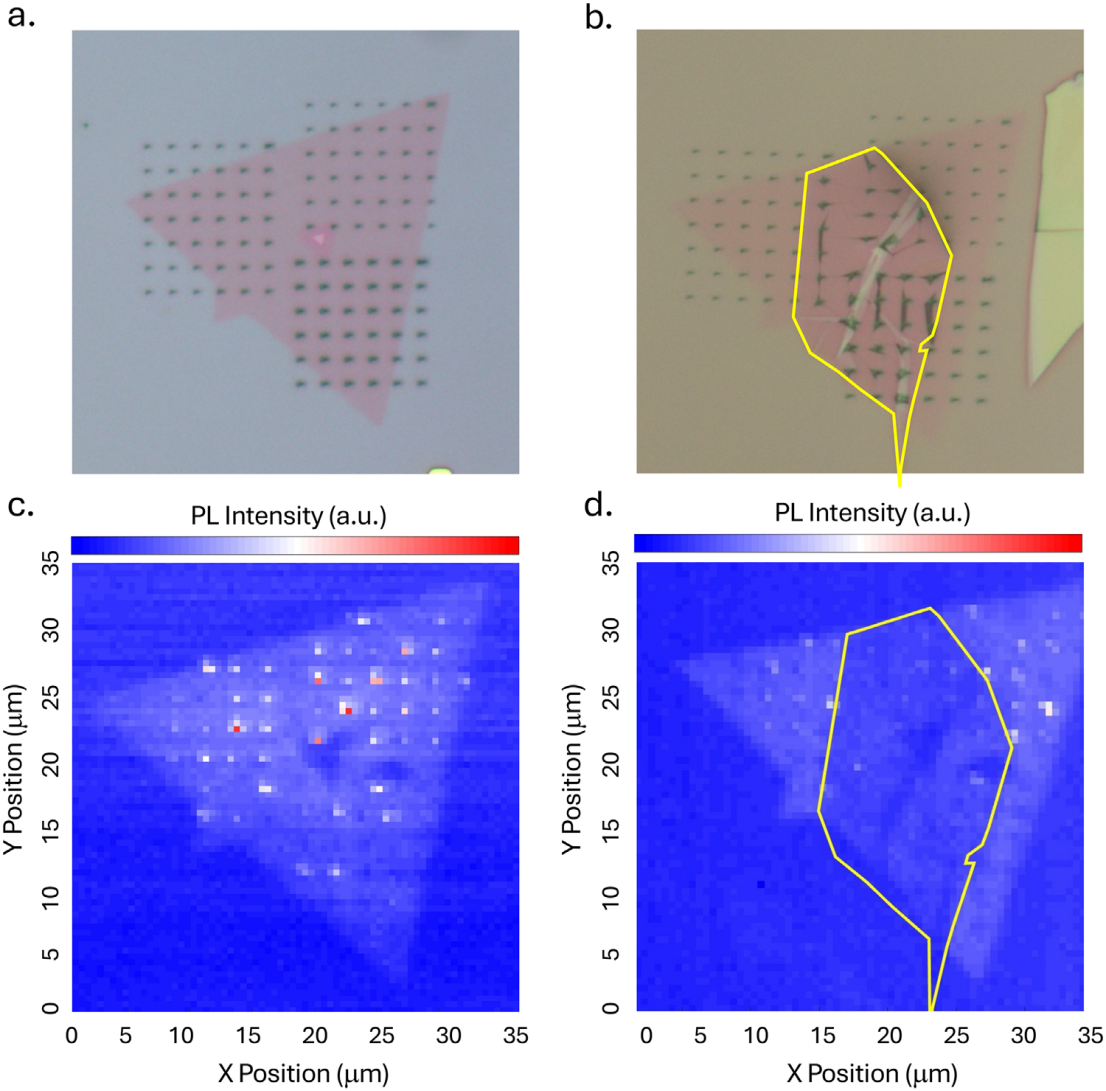
Results of a heterostructure with graphite transferred directly on top of functionalized monolayer WSe_2_. a) OM of a functionalized monolayer. b) OM of this same monolayer with graphite (outlined in yellow) transferred on top. c) Intensity map showing maximum peak intensity of the functionalized monolayer without graphite. d) Intensity map of the functionalized monolayer with graphite (outlined in yellow) transferred on top. Here, the graphite suppresses nearly all emission from both flat and strained areas. Localized emission from indents is very weak and disappears by ∼10*K*.

To definitively test whether the improved purity and elevated temperatures of Section 2.1 are due to the covalent interaction of diazonium on graphite specifically, two control tests were performed. The precedent of diazonum functionalization of WSe_2_ was established with exfoliated monolayers. Since our results were obtained with CVD WSe_2_, which has a higher defect density on average,^[^ ^33^, ^34^ ^]^ the 4‐NBD functionalization of the latter is tested alone to ensure that the effects are consistent between materials. Indeed, the modification is consistent with that observed with exfoliated material; emission from flat regions of the monolayer is suppressed, but SPE on the strain array remains (Figures S1 and S2, Supporting Information). This further demonstrates that 4‐NBD functionalization is a versatile modification method, effectively enhancing spectral isolation and purity in both exfoliated and CVD monolayers, using both nanopillars and nano‐indented strain arrays. However, these properties cannot be sustained beyond the typical temperature scales, even with the varied defect environment of CVD monlayers. As another test, the same WSe_2_ had graphite transferred onto it after functionalization was performed, and thus oligomerization had already occurred. As seen in **Figure** **4**, graphite suppresses the emission in both the flat and strained parts of the flake, as it does on WSe_2_ alone.^[^ ^11^ ^]^ The enhanced intensity and elevated temperatures cannot be replicated when the non‐covalent functionalization process occurs with WSe_2_ rather than a covalent functionalization with graphite (Figure 4c,d). This set of characterization techniques illustrates a clear picture of how the 4‐NBD functionalization occurs in the experimental heterostructure. Thin graphite (∼1.6 nm) is flexible and in close contact with the flat areas of WSe_2_ after the nano‐squeegee process. The nitrophenyl groups covalently functionalize the top graphene surface, evidenced by stability calculations and Raman spectroscopy showing new covalent bonds. Physisorption of oligomers to WSe_2_ or graphite as a cause is ruled out, demonstrating that high‐purity and elevated temperature observations can only be produced when the graphite surface is covalently functionalized.

### 2.3 Modeling

**Figure 5:**
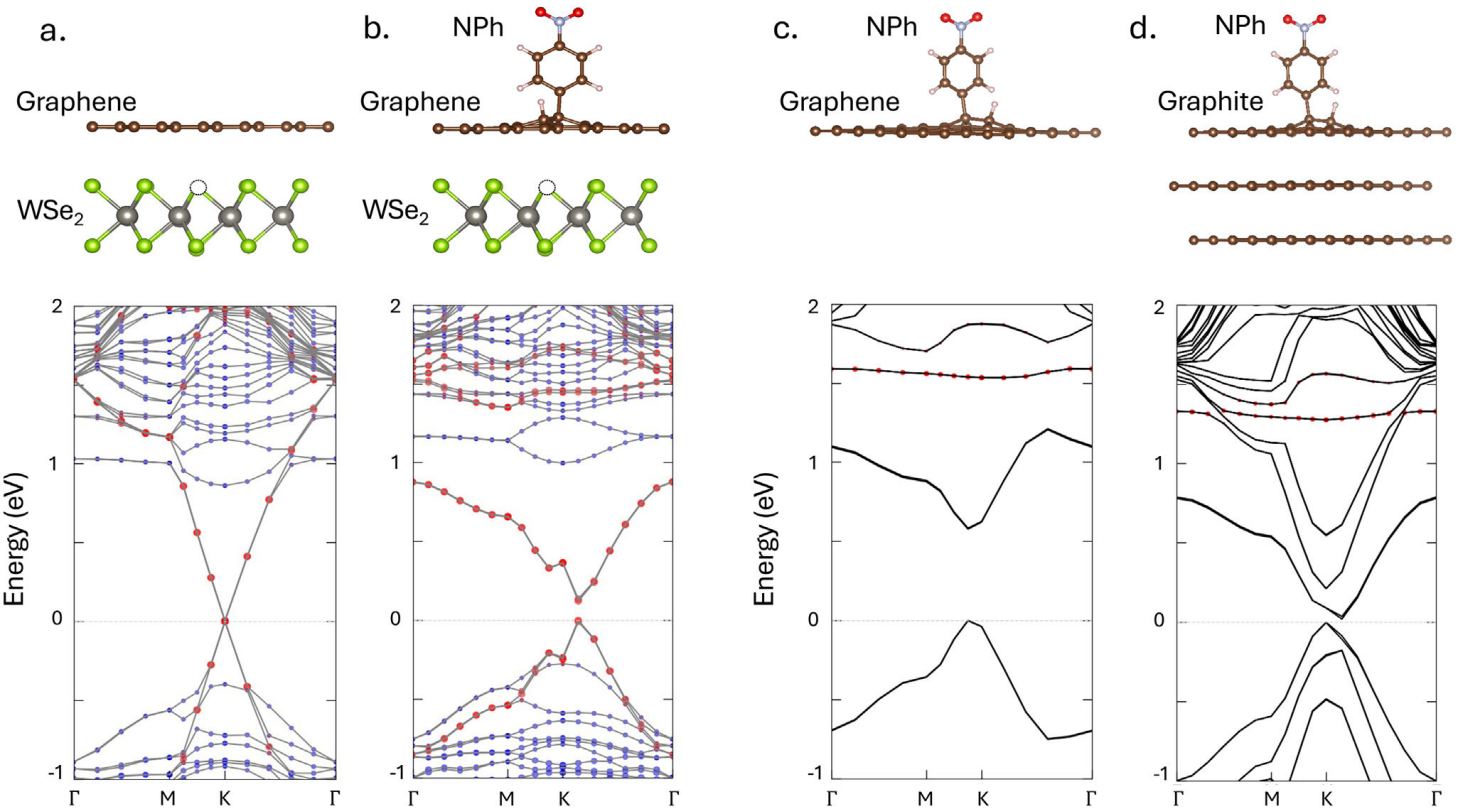
The electronic band structures of experimental systems in this work. In the band structures of (a) and (b), energy levels of graphene character are represented with red dots, whereas energy levels of WSe_2_ character are represented with blue dots. In the band structures of (c) and (d), the graphene/graphite energy levels are solid black lines, and the energy levels from nitrophenyl are dotted red. a) Monolayer graphene on top of monolayer WSe_2_ that has a single selenium vacancy (dotted black circle). In this heterostructure, the graphene is a semi‐metal as usual. Furthermore, it shows that the crossing of the graphene energy band with the conduction band minimum (CBM) of WSe_2_ will destabilize the CBM. b) Functionalized monolayer graphene on top of WSe_2_ that has a single selenium vacancy. Here, a small gap has been introduced to the graphene energy levels. Additionally, the crossing of the graphene and WSe_2_ CBM is now avoided, which stabilizes the CBM and prevents non‐radiative internal conversion. c) Band structure of functionalized graphene; here, a relatively large bandgap of 0.5 eV is opened by functionalization. d) Band structure of functionalized three layer graphite. Even with one nitrophenyl covalently bonded to the top surface, the semimetal structure is broken.

Chemical functionalization with aryl diazonium groups is an established tool to tune 2D materials, including graphite,^[^ ^24^, ^25^, ^35^, ^36^ ^]^ but this modified graphite has yet to be leveraged for enhancing the properties of quantum emission. With experimental and computational evidence that 4‐NBD covalently functionalizes the top graphite surface of our heterostructure, density functional theory (DFT) calculations were performed to demonstrate how the altered graphite affects the electronic band structure of the system. For this, the model assumes that graphene/graphite is in contact with WSe_2_, which is discussed further at the end of this section. **Figure** **5** shows the band structure of defective monolayer WSe_2_ in a heterostruture with pristine and functionalized graphene, as well as the difference between functionalized graphene and functionalized graphite. It is well established that 1L WSe_2_ is a direct bandgap semiconductor,^[^ ^37^, ^38^ ^]^ with midgap defect states introduced by common defects, such as selenium vacancies. Defect‐bound transitions through the resulting energy levels are proposed as the source of SPE.^[^ ^10^, ^18^, ^39^, ^40^, ^41^, ^42^, ^43^, ^44^ ^]^ Pristine graphene exhibits its characteristic Dirac cone at the K point,^[^ ^45^, ^46^ ^]^ as seen in Figure 5a, where red dotted lines represent energy levels of graphene character. However, when new chemical bonds are introduced on the surface, a bandgap opens in the graphene energy levels such that there is no longer a Dirac cone at the K point (Figure 5b). The graphene now has a small bandgap that resides within the WSe_2_ bandgap. This has relevant implications for the resulting behavior between layers. First, the fact that graphite is no longer a semi‐metal means that carrier mobility has been reduced. Higher degrees of chemisorption reduce the carrier mobility of both electrons and holes in graphene,^[^ ^25^, ^36^ ^]^ which localizes carriers at these sites. Furthermore, the lower of the two functionalized graphene energy levels is now energetically higher than the valence band maximum (VBM) of WSe_2_ and below the Fermi energy. This means that the energy levels can interact optically with WSe_2_ defect states rather than acting purely as an ultrafast charge transfer mechanism as outlined in previous work.^[^ ^11^ ^]^ Figure 5c,d demonstrates the difference between functionalized single layer graphene vs. thin graphite. The bandgap of functionalized graphene alone is larger and consistent with previous calculations,^[^ ^36^ ^]^ but the electronic structure is still altered even with three layers of graphene, which is closer to our experimental system of approximately 5 layers.

Here, it is important to note that many factors affect the precise magnitude of both WSe_2_ defect transition energy gaps and the functionalized graphite bandgap. First, since the exact defects and mechanisms responsible for SPE are still largely debated,^[^ ^10^, ^18^, ^39^, ^40^, ^41^, ^42^, ^43^, ^44^ ^]^ each model produces a different story of SPE origin and how that is inferred from electronic structure calculated by DFT. Next, it is critical to consider the role of mechanical strain. Strain is an experimental necessity for observing SPE in this material, as previously discussed. But strain also alters other optoelectronic properties of semiconductors and can modulate the bandgap of WSe_2_, generally red‐shifting its emission.^[^ ^38^, ^47^, ^48^, ^49^, ^50^ ^]^ The properties of SPE are very sensitive to microscopic strain variations. As such, the precise energy gap of any single SPE transition in a given strain environment is very difficult to predict.

Another factor to consider is the degree of covalent functionalization present on the graphene or graphite surface. In this work, the model has only one out of every 50 carbon atoms covalently attached to a nitrophenyl group, and this is enough to open a small bandgap. However, the local density of covalent bonds can be higher or lower since the diazonium is likely not evenly distributed across the graphite surface. If the concentration of nitrophenyl bonds is locally higher or lower, the magnitude of the bandgap will increase or decrease, respectively. In this regard, strain plays another important role. The density of covalent functionalization of 4‐NBD on graphite depends largely on two factors: material thickness and amount of strain.^[^ ^25^ ^]^ As demonstrated by our Raman spectroscopy, the degree of chemisorption between diazonium and graphite increases with decreasing layer number (Figure 3a). Furthermore, strain on the graphite surface can help locally increase the density of covalent bonds.^[^ ^25^ ^]^ Although the graphite in these samples is not indented itself, it does not lie completely flat on the indented WSe_2_. Where the schematic in Figure 1a shows a simplified device architecture, an accurate nano‐indent profile is shown in Figure S8 (Supporting Information). The indents are asymmetric and have “shoulders” instead of a flat opening. When few‐layer graphite is nanosqueegeed onto this profile, it conforms to and adopts some surface curvature as well. This, in turn, can increase the degree of covalent functionalizaton on the graphite at that site. Previous work utilizing nano‐indentation in WSe_2_ identifies the “shoulder ring” as the spatial location of SPE.^[^ ^9^ ^]^ Given this precedent and the effect of strain on graphite functionalization, it is the likely spatial location of our observed SPE phenomenon, although the current experiments do not provide direct evidence of the location of activated defects at these length scales.

Given all these considerations, our model represents the underlying principles at work in the system and encapsulates the key features of the sample: defects create midgap states in WSe_2_, and covalently functionalizing graphene or graphite opens a small bandgap within that of pristine WSe_2_. The magnitudes of each can vary, leading to complex interactions or even potential resonance between energy levels, and such interacting midgap states increase the binding energy of single‐electron transitions. This model is highly relevant in the “shoulders” where the heterostructure layers are in good contact and the SPE likely originates.^[^ ^9^ ^]^ The strain profile, heterostructure layer coupling, and efficacy of functionalization are not uniform across the indentation. The observations highlight that all three (heterostructure, strain, and functionalization) are required for high‐temperature SPE to form, suggesting that the DFT model used here is a reasonable configuration for understanding the observations. Further exploration of spatial profiles of all these features motivate promising directions for future work.

## 3 Discussion

The system presented here is complex, with crystalline defects, vdW material layer interactions, and covalently bonded molecules. From this combination, high‐purity SPE at elevated temperatures was achieved. Given this complexity, it is helpful to review the constituent materials systems that have been investigated individually. From the literature, it is understood that SPE can be observed in strained areas of plain WSe_2_,^[^ ^2^, ^3^, ^4^, ^5^ ^]^ although qualities such as line width and purity are variable, and the typical temperature limit to observe said SPE is around *T* = 15 K. The effect of diazonium functionalization on strained WSe_2_ was studied by Utama et al., who demonstrated that physisorption of nitrophenyl oligomers results in resonant charge transfer, isolating SPE of better spectral purity.^[^ ^10^ ^]^ We demonstrate that this is consistent between exfoliated and CVD‐grown monolayers. However, this system is also limited to around *T* = 15 K. Similarly, the effects of layering graphite in a heterostructure with strained WSe_2_ were investigated by Chuang et al., who demonstrated that the semi‐metal structure of graphite induces ultrafast charge transfer with WSe_2_, suppressing classical emission background and improving SPE purity.^[^ ^11^ ^]^ Once again, this system is also limited to the familiar temperature scales. In contrast, the system presented here contains all of these material components, yet the results are entirely unique, with very high purity SPE maintained to around *T* = 100 K. Experimentally, we deduced that those results come from covalent interactions between diazonium and graphite, and that the maximum achievable temperature is correlated with the graphite thickness. In the sample with thicker graphite, the D/G ratio was not high enough to be detected by Raman spectroscopy, and the elevated SPE temperature was only observed at one indent location, likely where the local density of bonded molecules was high enough to alter the material. With thinner graphite, the D/G ratio was clearly detected with Raman, and the yield of high temperature emitters across the strain array was correspondingly high. Knowing this, DFT modeling was performed to understand what makes functionalized graphite in this heterostructure unique from the point of view of electronic structure. From this we see that functionalizing graphene or graphite causes energy gaps to be introduced into what are typically continuous semi‐metal energy levels, which significantly alters the overall heterostructure. Computationally, a greater degree of covalent functionalization leads to a larger graphene/graphite band gap. Experimentally, the greater the degree of covalent functionalization, the higher the maximum SPE temperature can be reached.

**Figure 6:**
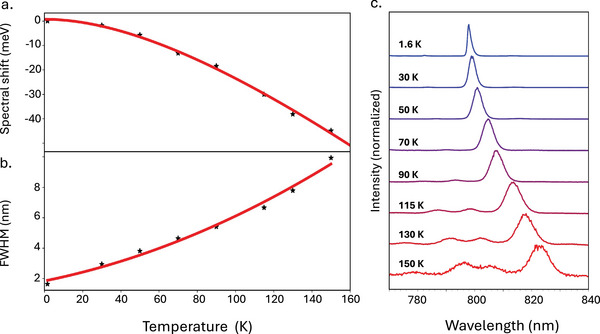
a) Spectral shift of SPE PL peak center with temperature. Data points are shown as black stars, and the Varshni fit line is shown in red (precedent for this fit established by Parto et al.^[^ ^18^ ^]^). b) Evolution of SPE linewidth as a function of temperature for the same source. Data points are shown as black stars, and the quadratic fit line is shown in red. c) PL spectra of the SPE with increasing temperature (spectra offset for clarity).

It is also helpful to discuss the results in the context of other reports of elevated SPE temperatures in 2D WSe_2_. Parto et al. demonstrated SPE at ∼150 K by irradiating WSe_2_ to induce defects that can have binding energies higher than normal.^[^ ^18^ ^]^ The SPE observed here have some quantifiable similarities, such as redshifting with increased temperature (**Figure** **6**a,c) and broadening linewidth (Figure 6b). Yet our approach operates on the distinct premise of using intrinsic defects in their typical densities, indicating that the mechanisms of these results are different. In another example, Luo et al. integrated WSe_2_ into plasmonic nanocavities^[^ ^17^ ^]^ to observe SPE up to 160 K. Strongly coupled systems such as optical cavities enhance light–matter interactions,^[^ ^51^ ^]^ with the Purcell‐enhanced ratio of radiative to non‐radiative recombination in nanocavity‐coupled WSe_2_ leading to higher accessible temperatures for SPE. The explanation in Ref. [17] based on enhanced quantum yield provides a possible interpretation of our work. As noted, the graphite bandgap can be resonant with WSe_2_ midgap states under the correct defect and strain conditions.

However, a notable difference in our work compared to the nanocavity‐coupled SPE is the high photon purity at elevated temperature. The SPE presented in Figure 2 and Figure S5 (Supporting Information) exhibit spectral broadening compared to their low‐temperature counterparts, with an average full width half maximum (FWHM) of ∼5 nm compared to <2 nm, respectively (Figure 6b). Generally, such broadening due to effects like phonon scattering and exciton‐phonon coupling^[^ ^52^, ^53^ ^]^ causes the purity of SPE to decrease when temperatures increase. As temperature increases, the non‐radiative relaxation rate also increases. In these prior realizations, the quantum yield decreases. For the purity of *g* ^(2)^(0) to decrease, additional radiative pathways must be excited beyond the relevant single‐photon transition. Broadening of the emission from non‐radiative processes at elevated temperature does not necessarily pollute the photon purity, although these are often correlated. Systems exist in which SPE are high purity at room temperature, even with broad linewidths,^[^ ^54^, ^55^ ^]^ yet this has not been true for TMDs thus far. In our case, the combination of the functionalization and the graphite heterostructure appears to sufficiently quench undesired radiative emission, even while allowing for broadening from non‐radiative emission, making it a unique result. The emitters presented here exhibit very high purity at *T* = 90 K even with spectral broadening, which means that scattering and other competing recombination mechanisms are not being introduced with increased temperature, as they are in other systems. To our knowledge, purity over 90% has not yet been reported in SPE beyond ∼10 K.^[^ ^7^, ^17^, ^18^ ^]^

In conclusion, we have demonstrated that diazonium functionalization is a broadly applicable and scalable method for improving the photon purity of SPE. More importantly, we have shown that SPE of very high purity can be sustained to *T* = 90 K in a functionalized WSe_2_/graphite heterostructure and observed with *g* ^(2)^(0) < 0.5 up to *T* = 115 K. Materials characterization, modeling, and control measurements suggest that this is caused by the covalent bonding between nitrophenyl and the graphite surface. DFT reveals that covalently attached nitrophenyl opens a bandgap in graphene and graphite that exists within the bandgap of WSe_2_ and localizes charge carriers. This resulting interaction with midgap states from defects creates bright, pure SPE that exceeds the typical temperature constraints of those qualities by roughly 80 K. Combined with prior reports, this result adds further evidence that chemical functionalization is a viable pathway for tailoring TMD optical properties beyond purity and can alter the fundamental mechanisms of a heterostructure when interacting covalently. This molecular and structural engineering of TMD defect emission demonstrates a new framework to control quantum photon sources that harnesses the standout benefits of this class of materials.

## 4 Experimental Section

### Material Growth

For CVD‐grown sample configurations, the monolayer WSe_2_ was synthesized on Si/SiO_2_ substrates featuring a 275 nm oxide layer, using quartz tube furnaces according to established procedures. To facilitate lateral growth, perylene‐3,4,9,10‐tetracarboxylic acid tetrapotassium salt molecules are deposited on the growth substrates. High‐purity metal oxide and chalcogen powders are employed as precursors, while a continuous flow of ultra‐high purity argon is maintained during the heating process to approximately 850 °C. Upon reaching the target temperature, ultra‐high purity hydrogen is introduced into the argon stream and sustained throughout the soak period and subsequent cooling to ambient temperature.^[^ ^56^ ^]^

### Sample Fabrication

Two types of sample configurations were carefully fabricated on the PMMA substrate for SPE generation. For both samples types, polymethyl methacrylate (PMMA) 950A4 was spun onto a clean SiO_2_ substrate and cured at 180 °C for 2‐3 minutes to obtain the 400nm thickness of PMMA in the SiO_2_ wafer. To obtain the first sample configuration (exfoliated 1L‐WSe_2_/PMMA) we directly exfoliate monolayers of WSe_2_ onto PMMA/SiO_2_; the WSe_2_ bulk crystals were purchased from HQ Graphene. To obtain the second sample configuration (CVD‐1L‐WSe_2_/PMMA), we use a solution‐assisted removal and transfer method to place CVD‐grown monolayer WSe_2_ on PMMA /SiO_2_.

The AFM nano‐indentation was then performed to generate the indentation array on both types of the sample configuration mentioned above, followed by the low‐temperature photoluminescence to confirm the existence of localized spectral features of the WSe_2_ indentation sites. Thin graphite flakes are then mechanically exfoliated onto either a PDMS patch or SiO_2_ substrate, subsequently identified by optical contrast and measured using AFM to determine thickness. A graphite flake of desired thickness is then directly transferred on top of the indented‐WSe_2_/PMMA substrate to partially cover the indented WSe_2_ array. All detailed steps of the sample fabrication can be found in previous studies.^[^ ^11^, ^16^, ^57^ ^]^

### Materials Characterization and Chemical Functionalization

Raman spectroscopy was performed under ambient and room temperature conditions using a Horiba XploRA Plus instrument and excitation with a 532‐nm laser (100x objective, NA 0.9). For chemical functionalization, the diazonium salt, 4‐nitrobenzenediazonium tetrafluoroborate (4‐NBD), was purchased from Sigma‐Aldrich (97%), stored at 4 °C and purified by recrystallization. An aqueous 5 mM solution of 4‐NBD was prepared no more than 15 minutes prior to use. The WSe_2_ or WSe_2_‐Gr samples were immersed in 5 mM 4‐NBD for 90 min in a glass scintillation vial that was shielded from light with aluminum foil. After immersion, the samples were rinsed with deionized (DI) water and dried with nitrogen flow.

### Low Temperature Optical Spectroscopy

Cryogenic optical spectroscopy and photon correlation measurements were performed in an AttoDry 2100 cryostat starting at *T* = 1.6 K and up to *T* = 150 K. High‐resolution spatial mapping was performed using Attocube piezoeletric nanopositioners in the *x* − *y* plane within the cryostat. Confocal spectroscopy was performed using a 0.82 NA, 100 × magnification objective with a 532 nm diode laser excitation. The diffraction‐limited spot size is estimated to be *D* = 1.22λ/NA ≈ 0.79 µ*m*. Light was collected with an optical fiber and sent to a 750‐mm focal length spectrometer (Andor Shamrock SR‐750) with a thermoelectrically cooled CCD camera (DU420A‐BEX2‐DD). A Hanbury‐Brown‐Twiss setup was used to measure the coherence. The signals were sent to an optical fiber with a 1 × 2 fiber splitter to direct equal signals to two avalanche photodiodes (APD; PicoQuant, τ‐SPAD‐100). To isolate light from the emitter of interest, a 10‐nm FWHM bandpass filter of the appropriate center wavelength was used. The raw photon coincidence data was used to calculate the second‐order intensity correlation function *g* ^(2)^(τ). Background correction was initially applied to check the final *g* ^(2)^(0) value. This correction only accounts for the background arising from dark counts on the APD from light contamination of the measurement environment. The background count rate was obtained from the APD with laser illumination blocked. For each SPE source presented in this work, the background correction did not alter the extracted *g* ^(2)^(0) value, and therefore the raw values are reported.

Emitter stability was measured in a Quantum Design Opticool cryostat; confocal spectroscopy was performed with a 532‐nm continuous wave laser excitation focused using a Nikon 100× 0.6 NA long working‐distance objective. The diffraction‐limited spot size is estimated to be *D* = 1.22λ/NA ≈ 1.08 µm.

### DFT Calculations

For DFT modeling, all calculations are performed using projector‐augmented wave (PAW)^[^ ^58^ ^]^ and generalized gradient approximation (GGA)^[^ ^59^ ^]^ with the Perdew, Burke, and Ernzerhof (PBE) exchange correlation functional as implemented in Vienna ab initio simulation package (VASP).^[^ ^60^ ^]^ To account for van der Waals (vdW) interactions, the DFT‐D3 correction proposed by Grimme was used.^[^ ^61^ ^]^ The cutoff energy for the plane wave function is 450 eV. For geometry optimization, a 4 × 4 × 1 Monkhorst‐Pack *k*‐grid^[^ ^62^ ^]^ is used, and for electronic structure calculations an 8 × 8 × 1 grid is used. The energy convergence criterion was set to 0.0001 eV and the whole system relaxed until the maximum forces on the atoms are lower than 0.001 eV/Å. Electronic band structures were calculated using Heyd‐Scuseria‐Ernzerhos hybrid functional (HSE06) coupled with spin‐orbit coupling (SOC).^[^ ^63^ ^]^ VASPKIT^[^ ^64^ ^]^ was used for some post‐processing band structure calculations. WSe_2_ was modeled with a 4 × 4 × 1 supercell and graphene was modeled using a 5 × 5 × 1 supercell. The heterostructure consists of WSe_2_ having a 4 × 4 × 1 supercell and graphene having a 5 × 5 × 1 supercell to minimize the lattice mismatch. When graphene is covalently functionalized with diazonium, for charge balancing, one H atom is attached to the neighboring carbon where the covalently bonded nitrophenyl group is attached.

## Conflict of Interest

The authors declare no conflict of interest.

## Author Contributions

S.C.G. performed optical spectroscopy and photon counting. A.D. performed chemical functionalization and Raman spectroscopy. M.K. executed first‐principles calculations. H‐J.C., S‐J.L., X.L., K.M.M., and M.I.B.U. fabricated monolayer WSe_2_ indentation and heterostructure samples. C.Z. performed spectral stability tests. S.C.G. prepared the manuscript with the input of all authors. G.C.S., T.J.M., M.C.H., B.T.J., and N.P.S. supervised the project.

## Supporting information

Supporting Information

## Data Availability

The data that support the findings of this study are available from the corresponding author upon reasonable request.
