# Supplementary material for: High‐Temperature Single‐Photon Emission From Covalently Functionalized van der Waals Heterostructures
Source: Adv Sci (Weinh). 2025 Oct 7;12(48):e11319. doi: 10.1002/advs.202511319 (PMC12752596; doi:10.1002/advs.202511319)
Supplement: Supplementary file 1 — Supporting Information [file ADVS-12-e11319-s001.pdf]

Supplemental Information: High-Temperature Single-Photon Emission from Covalently Functionalized van der Waals Heterostructures

S. Carin Gavin,[1] Hsun-Jen Chuang,[2] Anushka Dasgupta,[3] Moumita Kar,[4] Kathleen M. McCreary,[2] Sung-Joon Lee,[2] M. Iqbal Bakti Utama,[3] Chunxi Zhou,[1] Xiangzhi Li,[5,6] George C. Schatz,[4] Tobin J. Marks,[3,4,7,8] Mark C. Hersam,[3,4,8,9] Berend T. Jonker,[2] and Nathaniel P. Stern[1,*]

[1]*Department of Physics and Astronomy, Northwestern University, Evanston, IL 60208, USA*
[2]*Materials Science and Technology Division, United States Naval Research Laboratory, Washington D.C. 20375, USA*
[3]*Department of Materials Science and Engineering, Northwestern University, Evanston, IL 60208, USA*
[4]*Department of Chemistry, Northwestern University, Evanston, IL 60208, USA*
[5]*Department of Physics, Stevens Institute of Technology, Hoboken, NJ 07030, USA*
[6]*Center for Quantum Science and Engineering, Stevens Institute of Technology, Hoboken, New Jersey 07030, USA*
[7]*Department of Chemical and Biological Engineering, Northwestern University, Evanston, IL 60208, USA*
[8]*Materials Research Center, Northwestern University, Evanston, IL 60208, USA*
[9]*Department of Electrical and Computer Engineering, Northwestern University, Evanston, IL 60208, USA*

CONTENTS

I. $g^{(2)}(\tau)$ Correlation measurements and Purity 2

II. 4-NBD Functionalization of CVD WSe_2 Monolayers 2

III. SPE from Functionalized Heterostructures 4

IV. Nano-indentation Profile 7

References 8

* n-stern@northwestern.edu

I. $g^{(2)}(\tau)$ CORRELATION MEASUREMENTS AND PURITY

The second order photon correlation function $g^{(2)}(\tau)$ characterizes the intensity correlation of two optical detectors with time separation τ. For the single photon detectors used here, g^2 measures the non-classical photon arrival correlations indicative of quantum emission from a single source. A coherent light source, such as a laser, has a flat $g^{(2)}(\tau) = 1$ for all values of τ. This is equivalent to the same probability of a photon detected on two detectors as if they were entirely independent measurement events. In contrast, quantum emission from a single source is anti-correlated; detection of a photon at one detector does not generally coincide with a detection at a second detector. The correlation function $g^{(2)}(\tau)$ exhibits an antibunching dip in the coincidence counts at $\tau = 0$ that recovers as τ increases. Here, the long-τ behavior is assumed to be uncorrelated and is normalized to unity. This procedure establishes the numerical value of $g^{(2)}(0)$, which is used for statements of the purity of an emitter. The purity P quantifies how close to zero the correlation function gets, often expressed as a percentage:

$$P = \left[1 - g^{(2)}(0)\right] \times 100\% \tag{1}$$

The lower the $g^{(2)}(0)$ value, the higher the purity of the single photon source. As an example, the work presented in the main text discusses SPE with photon purity of over 90% because the $g^{(2)}(0)$ values are less than 0.1. A of $g^{(2)}(0) < 0.5$ is sufficient to conclude that the emitted light comes from a a single source. This condition is met by all SPE sources discussed in this work. As mentioned in the Methods, corrections to $g^{(2)}$ measurements for the Poissonian background dark counts are negligible on the scales presented.

To extract the recombination lifetime from the $g^{(2)}(0)$ measurements, they were fitted as a two-level system according to the equation, where τ_1 is the recombination time fitted to the anti-bunching dip width:

$$g^{(2)}(\tau) = \left[1 - (1 - g^{(2)}(0))\right] \cdot e^{-\frac{|\tau|}{\tau_1}} \tag{2}$$

Since the measurements are confirmed to be in a low-power limit, it is approximately equal to the excited-state lifetime.

II. 4-NBD FUNCTIONALIZATION OF CVD WSe_2 MONOLAYERS

Spectroscopy and photon correlation measurements were performed on CVD-grown monolayers treated with 4-NBD to compare with previous results on exfoliated material. Figures 1b,c show the qualitative difference on emission made by functionalization. Morphologically flat areas dominant the emission intensity prior to functionalization, whereas afterwards most of the excitonic complex on these areas is quenched. Localized emission remains in the strain array. Figure 2 shows a sampling of $g^{(2)}(\tau)$ measurements of the localized emission. Before functionalization, it is difficult to isolate any emitter to assess its quantum behavior, and those that are measured have a $g^{(2)}(0)$ above the threshold to be considered SPE. Afterwards, many more indents have isolatable emitters, and their $g^{(2)}(0)$ are much lower on average, although the precise value does vary with strain morphology, with indents slightly better and natural strain from a monolayer wrinkle slightly worse. It is noted that this set of $g^{(2)}(\tau)$ measurements is a representative sample. Because the CVD monolayers are large and have many indent sites, many more emitters can be assessed.

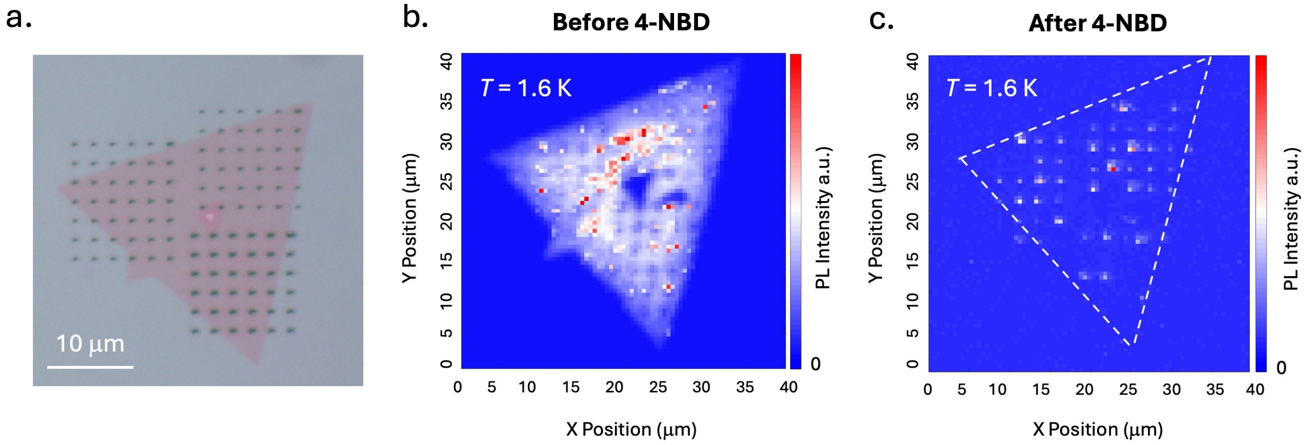

FIG. 1. Diazonium functionalization selectively preserves emission at strained regions of 1L WSe_2. (a) Optical image of the monolayer showing three arrays of nano-indents.(b) Spectral map of the monolayer at $T = 1.6$ K. Emission from indents here is relatively weak compared to the bright exciton fine structure and defect emission on areas between indents. After functionalization, emission is quenched on flat areas of the flake, leaving sharp peaks from the indent grids. A white dashed line outlines the monolayer area that was quenched.

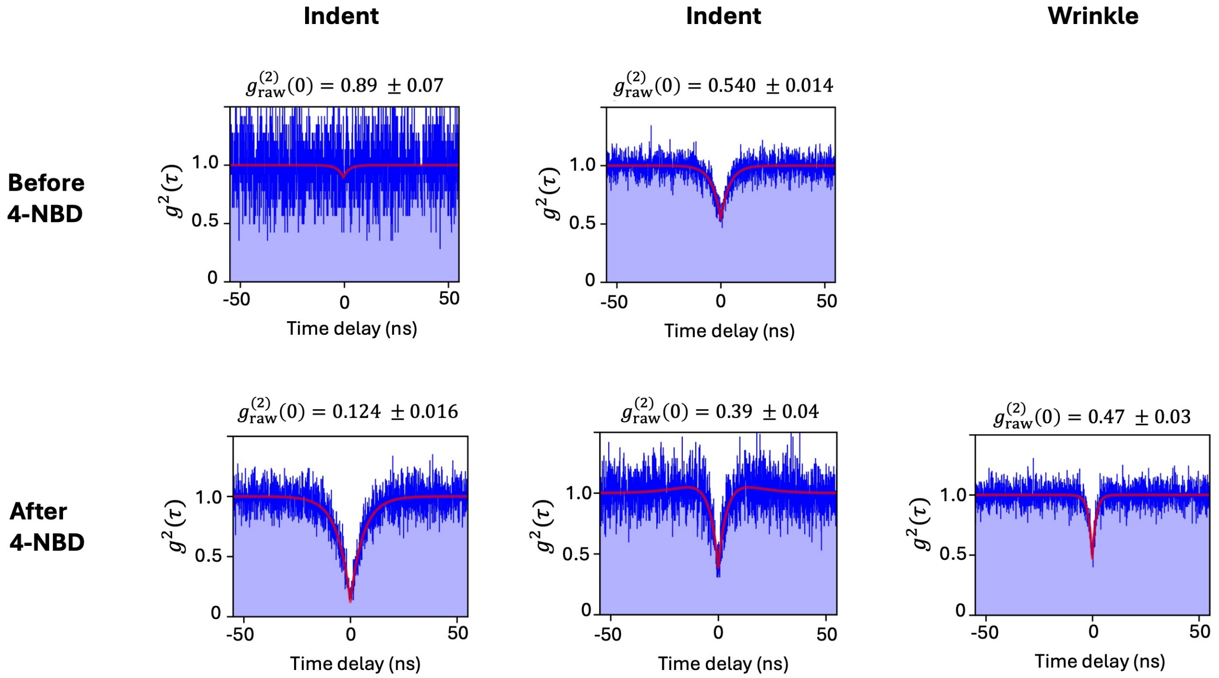

FIG. 2. Comparison of localized emission from CVD WSe_2 in Figure 1 before and after 4-NBD functionalization. Prior to 4-NBD, emitters are difficult to isolate for measurement, and their average purity is poor. After functionalization, more isolated emitters are available on both indents and naturally occurring strain such as wrinkles. Although $g^{(2)}(0)$ values on these emitters vary, they are lower on average and all within the threshold of < 0.5 to be considered a single photon source.

III. SPE FROM FUNCTIONALIZED HETEROSTRUCTURES

Figure 3 presents a histogram of emitters that are candidate SPE from our functionalized heterostructure between temperatures $T = 1.6$ K (blue) and $T = 90$ K (red). Spectra from the highest quality squeegeed WSe_2/graphite area were assessed, which presents a total of 24 indentation sites. An emitter in the histogram is set by two conditions: the peak must have a FWHM of less than or equal to that set forth for SPE from the fits presented in Figure 6. The maximum peak intensity must also be located at 780 nm or above in the spectrum, since those meet the physical requirements to last up to high temperatures. At $T = 1.6$ K, 17 emitters meet the SPE qualifications: peaks with a FWHM of less than 2 nm that are located beyond 780 nm in the spectrum. At $T = 90$ K, 14 emitters meet SPE qualifications: peaks of FWHM less than 6 nm and located beyond 780 nm. Since $g^{(2)}(\tau)$ measurements could not be performed on every emitter, these properties serve as a proxy to predict the yield of SPE at high temperatures. The baseline of this yield is the number of qualifying emitters at low temperature rather than the total number of strain sites, since it is possible based on monolayer and indent quality that no SPE would be present at all at any given strain site, which would automatically lower the yield at $T = 90$ K.

To assess SPE spectral wandering and jittering in the heterostructure, spectra were continuously collected for an emitter at $T = 1.6$ K. Figure 4 shows the trend of a representative emitter for 100 seconds. The average change is in line with that of SPE in related diazonium functionalization and heterostructure design systems [1, 2].

Figure 5 shows additional emitters at $T = 90$ K from the heterostructure discussed in the main text (Figure 2). Many of the indents from the nano-squeegeed area sustain localized emission at this temperature. Figure 5a shows four such locations, with maximum peak intensity between 600 and 1200 counts per second. The wavelength range emitters is also notable; as discussed in the main text, higher-energy emission is suppressed, while lower energy emitters, typically > 780 nm, selectively last up to the higher temperatures. An additional measurement $g^{(2)}(0)$ is shown in Figure 5b for the yellow spectrum in (a). Even for the weakest of the four emitters shown, the purity is greater than 90% and the signal-to-noise ratio is excellent. In total, two SPE are confirmed with $g^2(\tau)$ at $T = 90$ K, and 12 other points are identified as candidate SPE for their spectral position and linewidth. From the pool of their low-temperature counterparts, approximately 80% of candidate SPE are maintained at high temperatures.

Figure 6 shows the full evolution $g^{(2)}(0)$ and lifetime values as a function of temperature for the emitter highlighted in the main text (Figure 2). Even up to $T = 115$ K, it is viable SPE of good purity and high count rate. Beyond this temperature. the intensity decreased and became crowded with other weak spectral features enough for the correlation measurements to no longer be practical.

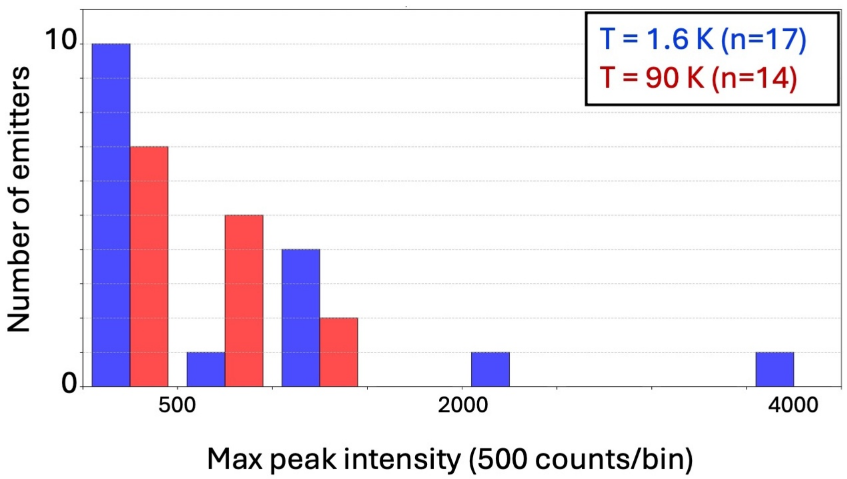

FIG. 3. A histogram of candidate emitters compared at $T = 1.6$ K (blue) and $T = 90$ K (red). Within the set parameters of spectral position and linewidth, 14 out of 17 candidate emitters at low temperature persist to higher temperatures.

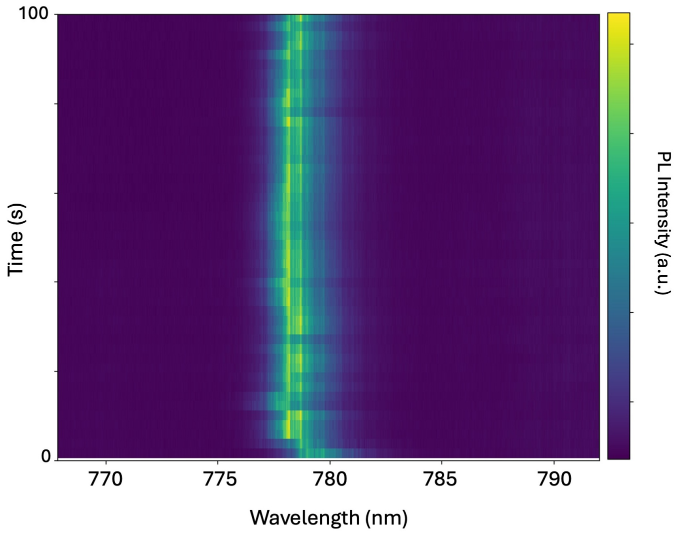

FIG. 4. Spectral stability of SPE from a functionalized graphite-covered indent at $T = 1.6$ K.

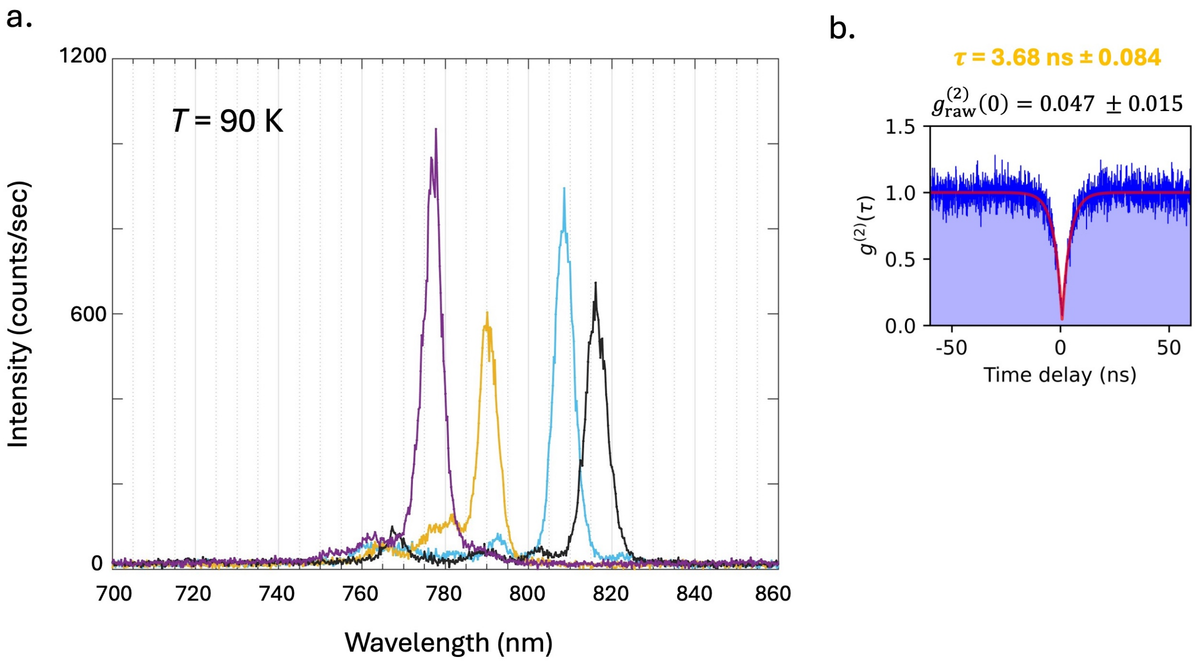

FIG. 5. (a) Spectra from four additional indent locations showing intense, low energy emission at $T = 90$ K. (b) $g^{(2)}(\tau)$ for the emitter shown in the yellow spectrum of (a), showing SPE of over 90% purity at this temperature.

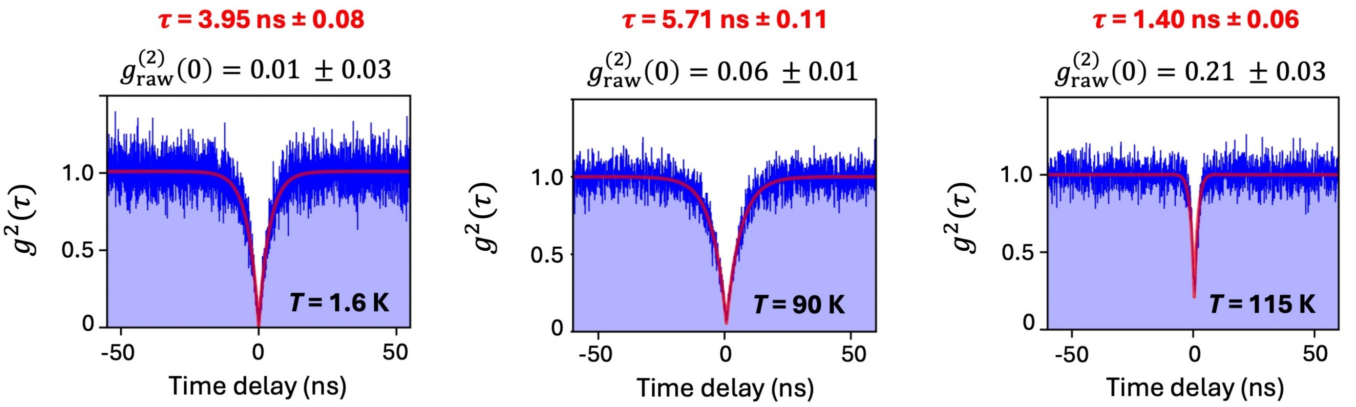

FIG. 6. Evolution of $g^{(2)}(\tau)$ with temperature of the emitter presented in Section II A of the main text.

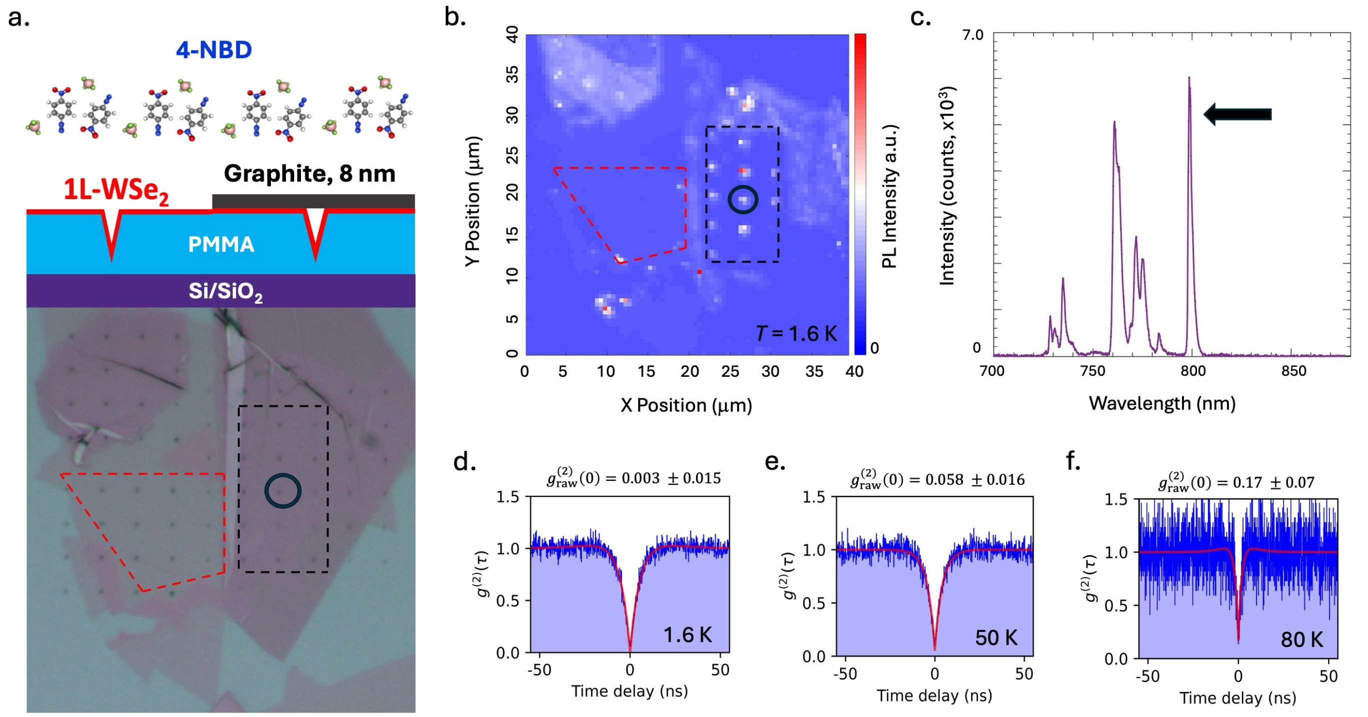

FIG. 7. Results of a heterostructure with very thick graphite layered onto indented, CVD-grown WSe_2. (a) Schematic of the heterostructure, which is identical to that presented in the main text other than the thickness of the layered graphite. (b) PL map of the heterostructure at $T = 1.6$ K. (c) Spectrum of the area circled in black from (b), with a strong, low energy emitter highlighted at 800 nm (black arrow). (d)-(f) $g^{(2)}(\tau)$ for the emitter shown in (c). This emitter is SPE with high photon purity at base temperature and at $T = 50$ K. By $T = 80$ K, the purity has decreased by an order of magnitude and the emitter intensity has dropped substantially, seen in the poor signal-to-noise ratio. The cutoff temperature for best purity is around 60-70 K, over 40 K less than the maximum temperature when the graphite is reduced to 1.6 nm.

IV. NANO-INDENTATION PROFILE

Figure 8 shows the AFM profile of nanoindents from our heterostructure both before and after graphite transfer. What is critical to note here is that the indents are not completely symmetric in x and y, nor are they flat on the surface. Rather, they have 'shoulders' on top that create additional strain and deform thin materials transferred on top of them. Furthermore, the indent itself is asymmetric between x and y and also around its own center. When graphite is transferred atop these indents, it conforms to these asymmetries and becomes microscopically strained. However, it still is not indented itself nor does it fully conform to the original indent shape, as seen in the AFM after the transfer of thin graphite that has been nanosqueegeed.

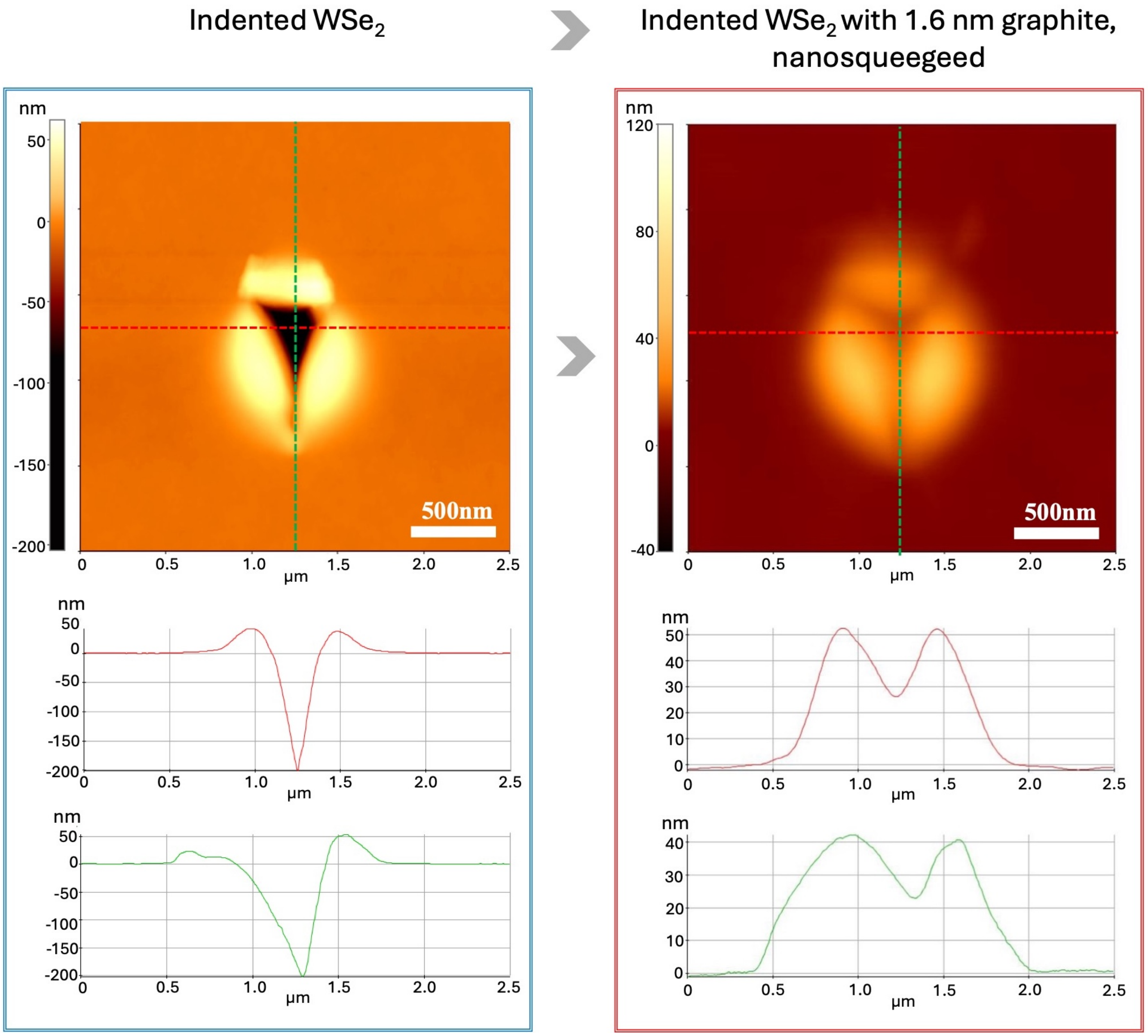

FIG. 8. The profile of a nano-indent measured by AFM before and after the transfer of graphite. The indent is not uniform along both axes. Rather, there are raised shoulders around the indent caused by the deformed polymer. When the WSe_2 is itself indented, the average depth of the indent is around 200 μm. Once thin graphite has been transferred on top and nanosqueegeed, it adopts some curvature within the existing indent profile and shoulders at the opening. In this way, graphite has become partially strained. However, it is not itself indented and does not reach the original full depth of the indented WSe_2.

[1] M. I. B. Utama, H. Zeng, T. Sadhukhan, A. Dasgupta, S. C. Gavin, R. Ananth, D. Lebedev, W. Wang, J.-S. Chen, K. Watanabe, *et al.*, Chemomechanical modification of quantum emission in monolayer WSe_2, Nature Communications **14**, 2193 (2023).
[2] H.-J. Chuang, C. E. Stevens, M. R. Rosenberger, S.-J. Lee, K. M. McCreary, J. R. Hendrickson, and B. T. Jonker, Enhancing single photon emission purity via design of van der Waals heterostructures, Nano Letters **24**, 5529 (2024).
